# Energy price instability and energy efficiency: Korea’s macroeconomic framework during the COVID-19 pandemic

**DOI:** 10.1371/journal.pone.0321793

**Published:** 2025-04-29

**Authors:** Renhong Wu, Yugang He, Zhuoqi Teng

**Affiliations:** 1 School of Management, Kyung Hee University, Seoul, Republic of Korea; 2 Department of Chinese Trade and Commerce, Sejong University, Seoul, Republic of Korea; 3 College of Business Administration, Henan Finance University, Zhengzhou, China; University of Salerno: Universita degli Studi di Salerno, ITALY

## Abstract

The pervasive effects of the COVID-19 pandemic in Korea on the daily lives of Korean citizens, as well as the nation’s economic and industrial landscape, cannot be understated. In this article, we explore the ramifications of energy price fluctuations, changes in energy efficiency, and shifts in monetary policy on the dynamic macroeconomic framework of the Korean economy during this unprecedented global crisis. Utilizing Bayesian estimation and impulse response functions, the study’s findings reveal that a surge in energy prices triggered a cascade of detrimental effects, including reductions in output, investment, employment, energy consumption, real wages, investments, real monetary holdings, and loan interest rates, while simultaneously elevating the deposit interest rate. Conversely, a positive shock to energy utilization efficiency engendered multiple favorable outcomes, such as greater output, consumption, employment, energy consumption, real wages, investment, and real money holdings, along with declines in deposit and loan interest rates. In the short term, a monetary policy shock precipitated an upswing in output, consumption, employment, energy consumption, investment, real money holdings, deposit interest rates, and loan interest rates, while exerting downward pressure on real wages. In sum, integration of these findings into the existing literature on the subject in the Korean context may significantly increase the depth and comprehensiveness of the discourse, improving our understanding of the multiple impacts of the COVID-19 pandemic on the nation’s economy.

## 1. Introduction

The COVID-19 pandemic has caused unparalleled challenges in the global economy; in this study, we conduct a rigorous and exhaustive investigation of its diverse and far-reaching implications [1–6]. South Korea, distinguished by its significant dependence on energy imports and extensive integration into the global market, is still grappling with the aftermath of the pandemic [7–11]. In the dynamic landscape of South Korea’s macroeconomy, we identify three key determinants of energy efficiency shocks: monetary policy, energy pricing, and the efficiency of energy use. Our results provide researchers and policymakers with a better understanding of the complex interplay among these elements as well as their cumulative influence on the stability and resilience of South Korea’s macroeconomy during this unprecedented global crisis.

In South Korea, the COVID-19 crisis impacted the energy sector significantly, as evidenced by the extraordinary volatility of oil prices in 2020 [12–15]. As a prominent energy-importing nation, South Korea’s macroeconomy is especially vulnerable to such price fluctuations. Moreover, remote work, diminished industrial activity, and evolving transportation-related behaviors have prompted reassessment of energy consumption and efficiency in South Korea [16–20]. Recent data highlighted a temporary reduction in greenhouse gas emissions during the pandemic [21–24]; subsequent shifts may or may not have enhanced energy efficiency and adoption of cleaner, more sustainable technologies in South Korea. In response to the pandemic, the Bank of Korea adapted by implementing unprecedented reductions in policy rates and unconventional monetary measures, such as bond purchases and liquidity provisions. We conduct a meticulous analysis of this swift and strategic response, examining its efficacy in navigating the extraordinary challenges presented by the COVID-19 pandemic.

Specifically, we investigate the impact of shocks related to monetary policy, energy pricing, and energy efficiency on South Korea’s macroeconomy. Employing sophisticated Bayesian estimation techniques and impulse response functions, we offer a comprehensive understanding of the intricate relationships among these critical factors. The findings reveal that positive energy price shocks have numerous effects, including decreased output, investment, employment, energy consumption, real wages, and real money holdings, as well as reduced lending rates and increased deposit rates. Conversely, a positive energy efficiency shock leads to increased output, consumption, employment, energy consumption, real wages, investment, and real money holdings and reduced deposit and lending rates. Our analysis underscores the importance of energy efficiency as a driver of economic growth and resilience, highlighting the need for targeted policy interventions that promote sustainable energy practices. Additionally, it demonstrates that monetary policy shocks trigger short-term increases in output, consumption, employment, energy consumption, investment, real money holdings, deposit rates, and lending rates while causing temporary declines in real wages. Our findings emphasize the complex interplay between monetary policy and the macroeconomic environment, and the need for policy transmission mechanisms to enable development of effective and well-calibrated responses. By shedding light on the intricate relationships among monetary policy, energy pricing, and energy efficiency, this paper contributes to the ongoing discourse on macroeconomic resilience and stability, informing development of robust policy frameworks to safeguard South Korea’s economic well-being in future crises.

This study makes three noteworthy contributions to the existing literature on the impact of the COVID-19 pandemic on Korea’s macroeconomic landscape: (1) by concurrently investigating three influential factors—shocks to energy prices, energy efficiency, and monetary policy—and their impact on a comprehensive array of economic indicators, we offer a robust and detailed understanding of the interplay among these factors and their collective effect on Korea’s macroeconomy during the pandemic; (2) utilizing cutting-edge methodologies, such as Bayesian estimation and impulse response functions, we analyze the effects of various shocks on Korea’s economic indicators in the context of the pandemic. Use of these advanced techniques bolsters the reliability of the results, thus increasing its significance within the existing body of literature; (3) we also provide crucial insights into the immediate ramifications of monetary policy shocks on Korea’s economic indicators, adeptly capturing the rapidly shifting dynamics of the macroeconomy during an evolving crisis. By juxtaposing these short-term outcomes with the longer-term implications of shocks to energy prices and energy efficiency, we introduce a valuable temporal dimension to the discussion, paving the way for a comprehensive understanding of policy interventions during times of crisis.

The paper is organized as follows: Section 2 examines relevant previous literature; Section 3 presents our model; Section 4 assesses and discusses the findings; and Section 5 describes the conclusions drawn from the study.

## 2. Theoretical framework and literature review

The three main factors examined herein, energy price shocks, energy efficiency shocks, and monetary policy shocks, are grounded in established economic theories that explain their impact on macroeconomic variables. Previous analyses of energy price shocks were based on classic supply and demand theory, which posits that fluctuations in energy prices affect the cost structures of firms and households. According to the theory of price transmission, an increase in energy prices raises production costs, leading to higher prices for consumers and potentially reducing output, consumption, and employment [25]. In this study, by contrast, we incorporate the theory of intertemporal substitution, which suggests that firms and households adjust their consumption and investment decisions over time in response to changes in energy price [26]. The theoretical basis of previous research on energy efficiency shocks has been endogenous growth theory, which emphasizes the role of technological advancements and efficiency improvements in driving economic growth [27]. According to this theory, enhanced energy efficiency reduces production costs and increases competitiveness, output, investment, and employment. In this study, we utilize rebound effect theory [28], which posits that gains in energy efficiency may lead to increased energy consumption, partially offsetting the benefits. Our analysis of monetary policy shocks is grounded in the New Keynesian framework, which highlights the role of central banks in managing economic fluctuations through interest rate adjustments [29]. We also integrate credit channel theory [30] to explain how changes in interest rates influence borrowing costs, investment decisions, and overall economic activity. Finally, the expectations hypothesis suggests that forward-looking behavior of firms and households plays a crucial role in the transmission of monetary policy [31].

Fluctuations in oil prices critically affect macroeconomic variables, influencing output, consumption, employment, and fiscal balances. They drive policy responses of central banks and governments, who attempt to maintain economic equilibrium amidst oil price-induced volatility. Jia et al. [32] used a computable general equilibrium model to evaluate the combined effects of the COVID-19 pandemic and crude oil prices on China’s low-carbon economy. They found that the pandemic-induced drop in oil prices adversely affected economic growth. Similarly, other studies [33–35] confirmed a substantial impact of the pandemic on carbon prices and economic stability in Europe. Further, Wu et al. [36] analyzed European carbon allowance futures prices using structural break tests and a cointegration approach; in that study, a negative relationship was found between increased carbon prices and short-term economic progress during the pandemic. Chien et al. [37] utilized wavelet analysis to show how rising COVID-19 intensity led to sharp decreases in energy prices, industrial output, and GDP. These findings were corroborated by other researchers [38–42] who underscored the pandemic’s significant impact on global energy prices and various economic variables.

Energy efficiency plays a pivotal role in sustainable economic development. Stern [43] elucidated the transmission mechanisms of energy efficiency shocks. Zhao et al. [44] provided empirical evidence of a positive relationship between improvements in energy efficiency and economic growth driven by productivity and technological advancements. Guha et al. [45] explored the spillover effects of energy efficiency on employment and investment and demonstrated the broad economic benefits of efficiency improvements. However, Van Binsbergen et al. [46] raised concerns about the “rebound effect,” in which efficiency gains can lead to increased energy consumption. Bento et al. [47] suggested that the relationship between efficiency shocks and macroeconomic indicators could involve bidirectional causality, a possibility that requires further scrutiny. Filippini and Hunt [48] highlighted the roles of governance and institutional quality in shaping the impact of efficiency shocks. Geller et al. [49] stressed the importance of incorporating energy efficiency into climate change mitigation strategies, while Linares and Labandeira [50] provided a comprehensive meta-analysis of the determinants and impacts of energy efficiency shocks, offering valuable insights for policymakers. Basher et al. [51] explored the effects of energy efficiency shocks on interest rates, identifying the transmission channels through which gains in efficiency impact monetary policy. Stojilovska et al. [52] outlined the potential socio-economic ramifications of energy efficiency shocks for distributional equity. Collectively, the seminal contributions of these previous studies enriched our understanding of the complex relationship between energy efficiency shocks and several macroeconomic variables. In the present study, we build upon this foundation, offering novel insights that contribute to the ongoing discourse and inform the development of context-sensitive policy.

Monetary policy is fundamental to economic stability. Li et al. [53] examined the credit channel mechanism of monetary policy, highlighting the importance of credit market frictions. Anwer et al. [54] used high-frequency identification techniques to analyze the relationship between monetary policy shocks and inflation, offering valuable insights for central banks. Caldara and Herbst [55] utilized a Bayesian approach to uncover the heterogeneous responses of macroeconomic variables to monetary policy shocks. Kamara and Koirala [56] focused on the labor market, revealing significant fluctuations in employment and wages due to monetary policy shocks. Cloyne et al. [57] explored the interaction between policy shocks and labor market rigidities, emphasizing context-sensitive interventions. Cumming and Hubert [58] demonstrated the role of housing markets in policy transmission, while Lee and Lee [59] examined the international transmission of policy shocks. Görtz et al. [60] stressed the importance of forward-looking elements in analyzing policy impacts. Tenreyro and Thwaites [61] investigated the asymmetric effects of policy shocks, and Clif [62] explored the link between policy shocks and financial stability.

Building on these foundational studies, our research employs Bayesian estimation and impulse response functions to analyze the impacts of energy price and efficiency shocks, as well as monetary policy shocks, on macroeconomic variables in Korea during the COVID-19 pandemic. By integrating these advanced methodologies, this study offers nuanced insights into the dynamic interplay of these factors, contributes to the ongoing discourse, and informs the development of context-sensitive policy.

## 3. Model

### 3.1. Household

Building upon previous seminal research [63], we posit that the economic framework under consideration embodies a prototypical household. Characterization of the utility function is articulated in the following manner:


$$\text{U}={\text{E}}_{t}\overset{\infty }{\underset{\text{t}=\text{o}}{\sum }}{\text{β}}^{t}\left[\frac{{\text{C}}_{t}^{1-\text{σ}}}{1-\text{σ}}-\frac{{\text{L}}_{t}^{1+\text{n}}}{1+\text{n}}+\frac{{\left(\frac{{\text{M}}_{t}}{{\text{P}}_{t}}\right)}^{1-\varrho }}{1-\varrho }\right].$$ (1)

In Eq (1), the utility function is represented by U, while E signifies the expectation operator. The discount factor is denoted by β, and consumption is encapsulated by C. The elasticity of consumption with respect to relative risk aversion is symbolized by σ, whereas L embodies labor input. The reciprocal elasticity of labor supply is given by n, and nominal monetary holdings are expressed as M. Furthermore, the price level is indicated by P, while the real balance’s elastic reciprocal is denoted by *ϱ*. This exposition lays the foundation for examining the budget constraints that an archetypal household encounters in a dynamic economic landscape.


$${\text{C}}_{t}+{\text{K}}_{\text{t}+1}+\frac{{\text{M}}_{t}}{{\text{P}}_{t}}-\frac{{\text{M}}_{\text{t}-1}}{{\text{P}}_{t}}+\frac{{\text{B}}_{t}}{{\text{P}}_{t}}={\text{W}}_{t}{\text{L}}_{t}+{\text{R}}_{t}^{\text{k}}{\text{K}}_{t}+\left(1-\text{δ}\right){\text{K}}_{t}+\frac{{\text{B}}_{\text{t}-1}}{{\text{P}}_{t}}{\text{R}}_{\text{t}-1}^{\text{b}}+{\text{D}}_{t}.$$ (2)

In Eq (2), K represents capital, while B signifies domestic one-period risk-free nominal bonds. The (gross) nominal interest is designated by ${\text{R}}^{b}$, and wages are denoted by W. Capital rental is symbolized by ${\text{R}}^{k}$, and the depreciation rate is represented by δ. Dividends are encapsulated by D. In unraveling the intricate relationship between lending and deposit interest rates, we draw upon various seminal works [64–66] in this study. The analysis unveils the dynamics underlying this vital aspect of financial systems.


$${\text{P}}_{t}{\text{W}}_{t}{\text{L}}_{t}={\text{ξ}}_{t}\text{ζ}{\left(\frac{{\text{Y}}_{t}}{\tilde{\text{Y}}}\right)}^{\text{τ}}{\text{B}}_{t}.$$ (3)

In Eq (3), ξ represents shocks to the lending interest rate, while ζ embodies the steady-state value of the loan-deposit ratio. Output is denoted by Y, with its steady-state value indicated by $\tilde{\text{Y}}$. The sensitivity of total loans to economic fluctuations is represented as τ. Drawing on previous methodologies [67–69], we postulate the existence of labor intermediaries within the economic structure. These intermediaries procure diverse laborers from households and subsequently supply them to production facilities, thereby functioning as a nexus between the two entities. Adopting the Calvo wage determination approach, as expounded in previous research [70–72], we employ a mechanism for households akin to the pricing method utilized by intermediate producers. Under this assumption, households can optimally adjust nominal wages upon receiving a stochastic “wage adjustment signal.” The probability of households receiving this signal in each period is denoted by 1−ψ. Consequently, the optimal nominal wage is adjusted to $\tilde{{\text{W}}_{t}}$, while the nominal wages of other households are modified in accordance with the preceding year’s inflation rate, effectively equating ${\text{W}}_{t}^{\text{n}}$ with ${\text{π}}_{\text{t}-1}{\text{W}}_{\text{t}-1}^{\text{n}}$. Upon achieving equilibrium among manufacturers, we determine the aggregate wage using the following equation:


$${\text{W}}_{t}^{\text{n}}={\left[\left(1-\text{ψ}\right){\left(\tilde{{\text{W}}_{t}}\right)}^{1-{\text{ν}}_{t}}+\text{ψ}{\left({\text{π}}_{\text{t}-1}{\text{W}}_{\text{t}-1}^{\text{n}}\right)}^{1-{\text{ν}}_{t}}\right]}^{\frac{1}{1-{\text{ν}}_{t}}}.$$ (4)

In Eq (4), ψ symbolizes the probability of the signal not being received, while $\tilde{\text{W}}$ represents the optimal wage. Furthermore, ${\text{W}}^{n}$ denotes the nominal wage, and π signifies inflation. The wage elasticity of labor demand is denoted by ν. When the nominal wage is adjusted, a representative household, j, has yet to receive the nominal “wage adjustment signal,” resulting in a wage level of ${\text{W}}_{\text{t}+\text{s}}^{\text{n}}$ during the t+s period. In scenarios where s exceeds one, the wage level is expressed as ${\text{W}}_{t}^{\text{n}}={\text{π}}_{t}{\text{π}}_{\text{t}+1}..{\text{π}}_{\text{t}+\text{s}-1}\tilde{{\text{W}}_{t}}$. Conversely, when s is equivalent to one, the wage level is represented by ${\text{W}}_{\text{t}+\text{s}}^{\text{n}}=\tilde{{\text{W}}_{t}}$. Integrating Eqs (1–4) yields the first-order condition for a representative household. We thereby capture the complex interplay among various economic variables and illuminate the dynamics governing household behavior.


$${\text{E}}_{t}\overset{\infty }{\underset{\text{s}=0}{\sum }}{\left(\text{βψ}\right)}^{\text{s}}\left[{\text{L}}_{\text{t}+\text{s}}{\text{C}}_{\text{t}+\text{s}}^{1-\text{σ}}\left(\frac{{\text{W}}_{\text{t}+\text{s}}^{\text{n}}}{{\text{P}}_{\text{t}+\text{s}}}-\frac{{\text{ν}}_{\text{t}+\text{s}}}{1-{\text{ν}}_{\text{t}+\text{s}}}\frac{{\text{L}}_{\text{t}+\text{s}}^{1+\text{n}}}{{\text{C}}_{\text{t}+\text{s}}^{1-\text{σ}}}\right)\right]=0.$$ (5)

### 3.2. Firms

Building upon the insights of previous research [73], we now distinguish between two discrete types of firms operating within the economic landscape. The first category encompasses firms engaged in the production of final goods, while the second category pertains to those involved in the production of intermediate products. The economy is presumed to consist of a continuum of intermediate firms operating in tandem. In a perfectly competitive market environment, the composition of the final product can be delineated as in Eq (6), which represents the intricate interdependencies among various economic agents and captures the essence of the production process.


$${\text{Y}}_{t}={\left(\overset{1}{\underset{0}{\int }}{\text{Y}}_{\text{i},\text{t}}^{\frac{\text{η}-1}{\text{η}}}\right)}^{\frac{\text{η}}{\text{η}-1}}.$$ (6)

In Eq (6), Y represents the final product, while ${\text{Y}}_{i}$ denotes the intermediate product. The substitution elasticity of intermediate products is symbolized by η. Given that the producer of the final product operates within a perfectly competitive market, its objective is to maximize profit contingent upon the prevailing prices of both the final and intermediate products. Our analysis elucidates this profit-maximization process, revealing the intricate dynamics at play in the manufacturer’s decision-making calculus.


$$\underset{{\text{Y}}_{\text{i},\text{t}}}{\max}{\text{P}}_{\text{t}}{\text{Y}}_{t}-\overset{1}{\underset{0}{\int }}{\text{P}}_{\text{i},\text{t}}{\text{Y}}_{\text{i},\text{t}}\text{di}.$$ (7)

In Eq (7), ${\text{P}}_{i}$ symbolizes the price of the intermediate product. Based on Eq (7), the output demand for the ${\text{i}}^{\text{th}}$ intermediate product producer can be represented by ${\text{Y}}_{\text{i},\text{t}}={\left(\frac{{\text{P}}_{\text{i},\text{t}}}{{\text{P}}_{t}}\right)}^{\frac{1}{\text{η}}}$. Moreover, in monopolistically competitive markets, manufacturers of intermediate products endeavor to optimize their operations according to certain principles. Eq (8) below highlights the strategies employed by firms to navigate complex market conditions.


$${\text{min R}}_{\text{t}}^{o}{\text{W}}_{t}{\text{L}}_{\text{i},\text{t}}+{\text{P}}_{t}^{\text{ec}}{\text{EC}}_{\text{i},\text{t}}+{\text{R}}_{i}^{\text{k}}{\text{K}}_{\text{i},\text{t}}.$$ (8)

In Eq (8), ${\text{R}}^{o}$ signifies the aggregate lending interest rate, ${\text{P}}^{\text{ec}}$ represents the price of energy, and EC encapsulates energy consumption. Drawing upon the findings of previous research [74–78], we denote the energy price shock by $\log\frac{{\text{P}}_{t}^{\text{ec}}}{{\tilde{\text{P}}}^{\text{ec}}}={\text{ρ}}_{\text{ec}}\log\frac{{\text{P}}_{\text{t}-1}^{\text{ec}}}{{\tilde{\text{P}}}^{\text{ec}}}+\log\frac{{\text{ω}}_{\text{t}}^{\text{ec}}}{{\tilde{\text{ω}}}^{\text{ec}}}+{\text{ρ}}_{{\text{ω}}^{\text{ec}}}\log\frac{{\text{ω}}_{\text{t}-1}^{\text{ec}}}{{\tilde{\text{ω}}}^{\text{ec}}}$. Furthermore, Eq (9) represents the budgetary constraints and fiscal challenges of a prototypical producer of intermediate products navigating this dynamic economic environment.


$${\text{Y}}_{\text{i},\text{t}}={\text{A}}_{t}{\text{K}}_{\text{i},\text{t}}^{\text{α}}{\text{L}}_{\text{i},\text{t}}^{\varpi }{\left({\text{ω}}_{\text{t}}{\text{EC}}_{\text{i},\text{t}}\right)}^{1-\text{α}-\varpi },$$ (9)

In Eq (9), A represents productivity, which can be construed as the aggregate amount of knowledge available within an economy pertaining to the “art” of production. The elasticity of production with respect to capital is denoted by α, while the elasticity of production with respect to labor is represented by *ϖ*. Shocks to energy efficiency are indicated by ω. Informed by previous research [79–82], we postulate that manufacturers of intermediate products employ the Calvo pricing mechanism to determine prices and maximize their real discounted profits. In each period, only a 1−ϕ proportion of intermediate manufacturers adjust their prices to achieve the optimal price p, while the remaining manufacturers calculate their indexed prices based on the previous year’s inflation rate, denoted by ${\text{π}}_{\text{t}-1}=\frac{{\text{P}}_{\text{t}-1}}{{\text{P}}_{\text{t}-2}}$. The degree of indexation is expressed as ${\text{P}}_{\text{i},\text{t}}={\text{π}}_{\text{t}-1}^{{\text{φ}}_{\text{p}}}{\text{P}}_{\text{i},\text{t}-1}$, with ${\text{φ}}_{\text{p}}\in \left[0,1\right]$. Consequently, the utility function, which maximizes the real discounted profit, can be articulated as follows:


$$\underset{\overset{\vee }{{\text{P}}_{t}}}{\max}{\text{E}}_{t}\overset{\infty }{\underset{j=0}{\sum }}{\left(\text{β}\phi \right)}^{\text{j}}{\text{λ}}_{\text{t}+\text{j}}\left[{\text{Y}}_{\text{i},\text{t}+\text{j}}\left(\overset{j}{\underset{m=1}{\prod }}{\text{π}}_{\text{t}-1}^{{\text{φ}}_{\text{p}}}\frac{\overset{\vee }{{\text{P}}_{t}}}{{\text{P}}_{\text{t}+\text{j}}}-{\text{MC}}_{\text{t}+\text{j}}\right)\right].$$ (10)

In Eq (10), λ denotes the Lagrange multiplier, MC denotes the marginal cost, and *ϕ* denotes the probability of signals not being received. Then, budget constraints are expressed as:


$${\text{Y}}_{\text{i},\text{t}+\text{j}}={\left(\overset{j}{\underset{m=1}{\prod }}{\text{π}}_{\text{t}-1}^{{\text{φ}}_{\text{p}}}\frac{\overset{\vee }{{\text{P}}_{t}}}{{\text{P}}_{\text{t}+\text{j}}}\right)}^{\frac{1}{\text{ς}}}{\text{Y}}_{\text{t}+\text{j}}.$$ (11)

In Eq (11), ς denotes the substitution elasticity between intermediate products. The first-order condition is as follows:


$$\overset{\vee }{{\text{P}}_{t}}=\frac{\text{ς}}{\text{ς}-1}\frac{{\text{E}}_{t}\sum_{j=0}^{\infty }{\left(\text{β}\phi \right)}^{\text{j}}{\text{λ}}_{\text{t}+\text{j}}{\text{Y}}_{\text{i},\text{t}+\text{j}}{\text{P}}_{\text{i},\text{t}+\text{j}}{\text{MC}}_{\text{t}+\text{j}}]}{{\text{E}}_{t}\sum_{j=0}^{\infty }{\left(\text{β}\phi \right)}^{\text{j}}{\text{λ}}_{\text{t}+\text{j}}\prod_{m=1}^{j}{\text{π}}_{\text{t}-1}^{{\text{φ}}_{\text{p}}}{\text{Y}}_{\text{i},\text{t}+\text{j}}}.$$ (12)

Therefore, the overall price level is as follows:


$${\text{P}}_{t}={\left[\left(1-\phi \right){\overset{\vee }{{\text{P}}_{t}}}^{1-\text{ς}}+\phi {\left({\text{π}}_{\text{t}-1}^{{\text{φ}}_{\text{p}}}{\text{P}}_{\text{t}-1}\right)}^{1-\text{ς}}\right]}^{\frac{1}{1-\text{ς}}},$$ (13)

These equations shed light on the complex interplay between various economic variables and their impact on firms’ decision-making processes.

### 3.3. Central bank

Drawing upon the insights of previous studies [83–85], we now posit that the monetary authority employs an augmented interest rate framework to govern deposit interest rates and influence output. Using this sophisticated approach, our analysis captures the strategies utilized by monetary policymakers to navigate complex economic conditions and maintain stability within the financial system.


$$\log\frac{{\text{R}}_{t}^{\text{b}}}{\tilde{{\text{R}}^{b}}}=\text{ι}\log\frac{{\text{R}}_{\text{t}-1}^{\text{b}}}{\tilde{{\text{R}}^{b}}}+\left(1-\text{ι}\right)\left[{\text{ρ}}_{\text{π}}\log\frac{{\text{π}}_{\text{t}}}{\tilde{\text{π}}}+{\text{ρ}}_{\text{y}}\log\frac{{\text{Y}}_{t}}{\tilde{\text{Y}}}\right]+\log\frac{{\text{ω}}_{\text{t}}^{r}}{{\tilde{\text{ω}}}^{\text{r}}}).$$ (14)

In Eq (14), ι denotes the smoothing coefficient of interest rates, ${\text{ρ}}_{\text{π}}$ denotes the reaction coefficient of interest rate rules to inflation, ${\text{ρ}}_{\text{y}}$ denotes the reaction coefficient of interest rate rules to output, ${\text{ω}}^{\text{r}}$ denotes monetary policy shocks, $\tilde{{\text{R}}^{b}}$ denotes the steady-state value of deposit interest rates, $\tilde{\text{π}}$ denotes the inflation rate, and $\tilde{\text{Y}}$ denotes the steady-state value of output.

### 3.4. Market clearing condition

The market clearing condition provides us with the following equation:


$${\text{Y}}_{t}={\text{C}}_{t}+{\text{K}}_{\text{t}+1}-\left(1-\text{δ}\right){\text{K}}_{t}+{\text{P}}_{t}^{\text{ec}}{\text{EC}}_{\text{t}}.$$ (15)

### 3.5. Data sources and parameter estimation

In this study, we employ parameters derived from two distinct sources: the first set originates from established literature in the South Korean context, while the second is obtained through Bayesian estimation using South Korean data. Our parameter calibration method aligns with those of He [86] for the elasticity of production with respect to capital (α =0.43), Kang and Suh [87] for the elasticity of production with respect to labor (*ϖ* =0.3), Lee and Song [88] for the discount factor (β =0.98), Choi and Hu [89] for the depreciation rate (δ =0.025), Pontines [90] for the sensitivity of total loans to economic conditions (τ =1.15), Kim [91] for the steady-state value of the loan-deposit ratio (ζ =0.56), Hur and Lee [92] for the probability of the price signal not being received (*ϕ* =0.75), Iwasaki et al. [93] for the probability of the wage signal not being received (ψ =0.75), and Hur and Rhee [94] for the price indexation degree (${\text{φ}}_{\text{p}}$ =0.3). Regarding Bayesian estimation, we utilize quarterly data on the Korean GDP and inflation spanning from Q1 2020 to Q3 2022. This period encompasses the outbreak of COVID-19; thus, it is a pertinent time frame for the purposes of our investigation. In line with previous research [95–97], we detrend the GDP and inflation using the Hodrick-Prescott filter, thereby isolating the cyclical components. The results of the Bayesian estimation are presented in Table 1.

**Table 1:** Results of Bayesian estimation.

Parameter	Definition	Prior distribution	Posterior mean	Confidence interval
σ	Intertemporal elasticity of substitution	Gamma [1, 0.25]	0.921	[0.901, 0.928]
n	Reciprocal elasticity of labor supply	Gamma [2, 0.25]	4.334	[3.214, 4.822]
*ϱ*	Elastic reciprocal of real balance	Gamma [2.5, 0.25]	2.438	[1.948, 2.816]
ι	Smoothing coefficient of interest rate	Beta [0.4, 0.3]	0.907	[0.745, 0.964]
${\text{ρ}}_{\text{π}}$	Reaction coefficient of interest rate rules to inflation	Gamma [1.75, 0.25]	1.769	[1.742, 1.827]
${\text{ρ}}_{\text{y}}$	Reaction coefficient of interest rate rules to output	Gamma [0.2, 0.1]	0.128	[0.078, 0.182]
${\text{ρ}}_{\text{ec}}$	Autoregressive parameter of energy price shock	Beta [0.5, 0.2]	0.613	[0.523, 0.659]
${\text{ρ}}_{\text{ω}}$	Autoregressive parameter of energy efficiency shock	Beta [0.5, 0.2]	0.487	[0.186, 0.831]
${\text{ρ}}_{{\text{ω}}^{\text{r}}}$	Autoregressive parameter of monetary policy shock	Beta [0.5, 0.2]	0.78	[0.712,0.893]
${\text{σ}}_{\text{ec}}$	Standard error of energy price shock	Inverse-gamma [0.01, inf]	0.048	[0.035, 0.069]
${\text{σ}}_{\text{ω}}$	Standard error of energy efficiency shock	Inverse-gamma [0.01, inf]	0.004	[0.002, 0.006]
${\text{σ}}_{{\text{ω}}^{\text{r}}}$	Standard error of monetary policy shock	Inverse-gamma [0.01, inf]	0.019	[0.016, 0.023]

## 4. Results

### 4.1. Effects of energy price shocks on key macroeconomic variables during the COVID-19 pandemic in Korea

**Fig 1:**
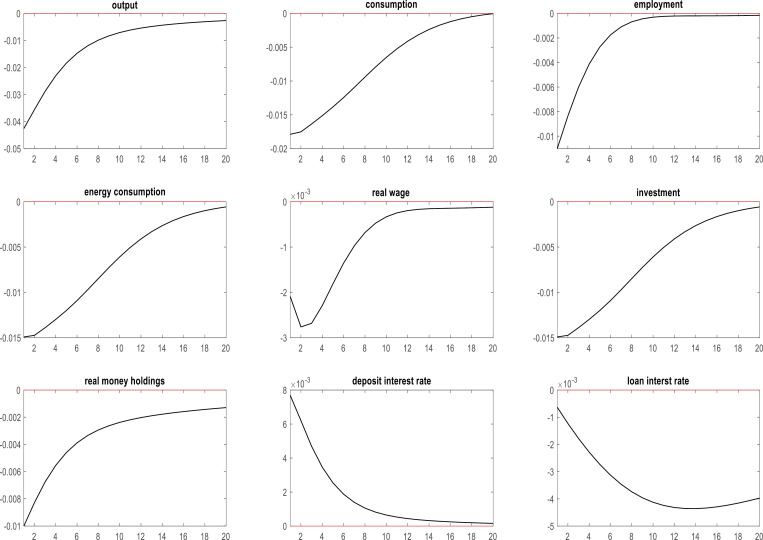
Effects of energy price shocks on key macroeconomic variables in Korea during the COVID-19 pandemic.

The COVID-19 pandemic wrought significant upheaval across the globe, leaving no nation unscathed, including South Korea. The effects of this unprecedented health crisis reverberated throughout the Korean economy; effects on raw material prices were particularly pronounced. According to data furnished by the Korean Ministry of Industry, Trade, and Resources, the price of coal utilized for power generation soared by 125.5% to $206.3 per ton during the pandemic, while the cost of imported liquified natural gas escalated by 68.4% to $534.9 per ton on a year-on-year basis. Considering these developments, the present study probes the ramifications of energy price shocks on pivotal macroeconomic indicators in the Korean context during the COVID-19 pandemic. Specifically, the analysis focuses on the interplay between energy price shocks and variables such as output, consumption, employment, energy consumption, real wages, investment, real money holdings, deposit interest rates, and loan interest rates. By systematically examining these relationships, we offer valuable insights into the intricate dynamics governing the Korean economy in a time of unparalleled global crisis. Fig 1 presents a comprehensive overview of the various repercussions of energy price fluctuations during the COVID-19 pandemic.

As illustrated in Fig 1, a positive energy price shock leads to a decline in both output and investment and an increase in energy consumption. One possible explanation for these results lies in the stringent measures imposed during the COVID-19 pandemic, when numerous firms were compelled to curtail their output or even cease operations due to regulations that restricted the gathering of individuals in South Korea [98]. The concurrent surge in energy prices, which augmented the cost of production-related activities, necessitated a reduction in corporate energy consumption and investment to maintain operational stability. The combination of output reduction and government-imposed restrictions in response to the pandemic contributed to a rise in unemployment. In addition, escalating energy prices drove up prices overall, thus eroding real wages for households in Korea. In tandem with the inflationary pressures, the general price increase prompted a corresponding decrease in real money holdings in Korean households. To counteract the detrimental effects on the Korean macroeconomy of soaring energy prices, the Central Bank of Korea implemented monetary policies such as increasing interest rates on residents’ deposits and reducing interest rates on corporate loans [99]. These measures helped to alleviate the erosion of wealth among Koreans and lowered operating costs for Korean firms, thus fostering a more stable macroeconomic environment during the COVID-19 pandemic.

### 4.2. Effects of energy efficiency shocks on key macroeconomic variables during the COVID-19 pandemic in Korea

**Fig 2:**
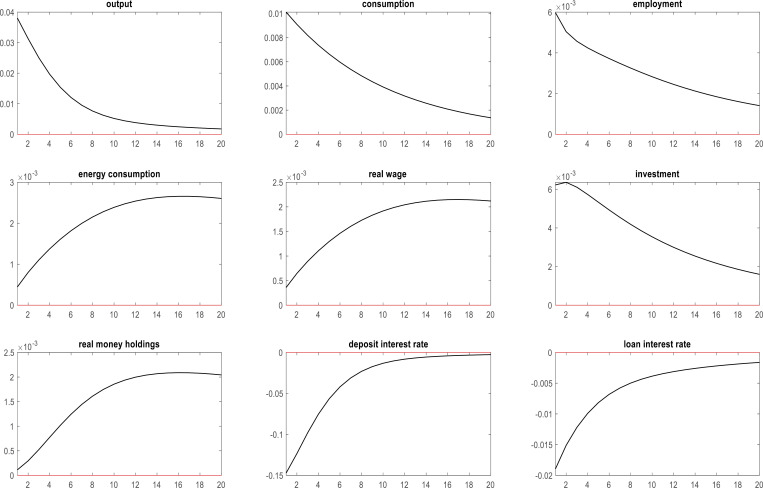
Effects of energy efficiency shocks on key macroeconomic variables in Korea during the COVID-19 pandemic.

Enhancing the efficiency of energy use may have mitigated the impact of the COVID-19 pandemic on the volatility of Korea’s macroeconomic indicators. In this section, we examine the repercussions of energy efficiency on key macroeconomic variables within the Korean context during the COVID-19 crisis. The interrelationships between energy efficiency and pertinent economic indicators provide valuable insights into the role of energy efficiency in promoting macroeconomic stability during challenging times. Fig 2 is a comprehensive depiction of the relationships between energy efficiency and these economic indicators during the COVID-19 pandemic.

As evidenced by Fig 2, a positive shock in energy efficiency contributes to an upswing in corporate output, investment, and energy consumption. This development proved advantageous for firms striving to sustain regular operations despite the adverse effects of the COVID-19 pandemic. In addition, a positive energy efficiency shock is associated with an increase in household consumption, employment, real wages, and real money holdings [100]. This enhancement in energy efficiency may have increased the availability of goods [101], which in turn increased consumption in Korean households, generated employment opportunities within related industries, and boosted the real wages of Korean citizens. Increased supply of goods led to a reduction in prices overall, thereby augmenting the real money holdings of the Korean population. Furthermore, a positive shock in energy efficiency triggered a decline in both deposit and loan interest rates during the pandemic. This observation has significant economic implications. Energy efficiency fosters a robust and resilient macroeconomic environment. By improving energy efficiency, policymakers and firms alike can stimulate economic growth, enhance employment prospects, and promote overall stability during periods of uncertainty such as Korea experienced during the COVID-19 pandemic.

### 4.3. Effects of monetary policy shocks on key macroeconomic variables during the COVID-19 pandemic in Korea

**Fig 3:**
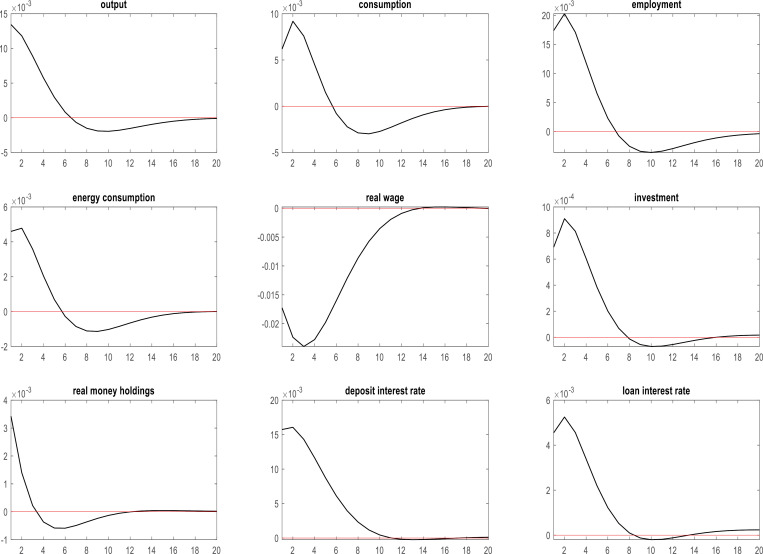
Effects of monetary policy shocks on key macroeconomic variables in Korea during the COVID-19 pandemic.

The conventional tools to measure monetary policy offer a clear picture of fluctuations in the Korean macroeconomy induced by the COVID-19 pandemic. In this subsection, we scrutinize the repercussions of monetary policy shocks on key macroeconomic variables in the Korean context during the COVID-19 crisis. The relationships between monetary policy and these economic indicators elucidate the ability of such policy measures to address macroeconomic volatility during unprecedented global challenges. Fig 3 presents a comprehensive overview of the relationships between monetary policy and these economic indicators in Korea throughout the COVID-19 pandemic.

As evidenced by Fig 3, monetary policy shocks augmented corporate output, investment, and energy consumption in the short term. This suggests that measures taken according to the Korean government’s monetary policy proved efficacious in the short run for mitigating macroeconomic turbulence in Korea triggered by the COVID-19 pandemic [102]. Additionally, shocks in monetary policy increased real monetary holdings of Korean households, stimulated consumption, and increased employment. However, they also induced a transient decline in real wages [103]. Other repercussions of monetary policy shocks included an upswing in both deposit and loan interest rates.

## 5. Conclusions

The economic implications of the findings of our study are numerous. Our findings underscore the interconnectedness of energy prices, output, investment, and employment in the context of a global health crisis, and the critical role of central banks in navigating the complex landscape of macroeconomic policy in response to exogenous shocks such as the COVID-19 pandemic. Our results may inform policy discussions and potential interventions aimed at bolstering resilience and fostering sustainable growth in the face of unprecedented global challenges. The insights gleaned from our analysis may serve as a valuable foundation for future research and policy discussions about energy efficiency and its broader economic ramifications. The results highlight the significance of carefully calibrated policy interventions to alleviate economic distress and foster stability in uncertain times, while also underscoring the need for ongoing monitoring and assessment of the long-term implications of such policy decisions.

The COVID-19 pandemic profoundly influenced various aspects of Korean society, causing substantial disruptions across the board. This study scrutinizes shocks in energy price, energy efficiency, and monetary policy and their effects on Korea’s economic indicators during the COVID-19 pandemic. Based on Bayesian estimation and impulse response functions, our analysis reveals that positive energy price shocks precipitated declines in output, investment, employment, energy consumption, real wages, investments, real monetary holdings, and loan interest rates while concurrently expanding deposit interest rates. Conversely, positive energy efficiency shocks escalated output, consumption, employment, energy consumption, real wages, investment, and real money holdings, while decreasing both deposit and loan interest rates. Lastly, monetary policy shocks instigated a short-term upsurge in output, consumption, employment, energy consumption, investment, real money holdings, deposit interest rates, and loan interest rates while simultaneously diminishing real wages. These findings underscore the interconnections among various shocks and the Korean macroeconomy during the tumultuous period of the COVID-19 pandemic.

The insights gleaned from the empirical analysis presented herein point to five pertinent policy recommendations.

(1) Policymakers should concentrate their efforts on devising and implementing strategies to stabilize energy prices, thereby attenuating the deleterious repercussions of energy price shocks on the economy. Tactical stockpiling, diversification of energy sources, and cultivation of alliances with dependable energy suppliers are all viable strategies.
(2) Governments should prioritize investments to enhance energy efficiency, as this factor was herein demonstrated to influence a range of economic indicators positively. Potential investments in energy-efficient technologies, promotion of energy-conserving practices, and establishment of incentive schemes to motivate households and businesses to adopt energy-efficient measures could all benefit Korean society.
(3) Central banks must exercise vigilance and adaptability in response to dynamic economic landscapes that emerge during crises such as the COVID-19 pandemic. This necessitates the tailoring of instruments of monetary policy, including interest rates and quantitative easing, to address the unique challenges of such crises, safeguard economic stability, and promote growth in both the short and long term.
(4) Due to the detrimental impact of economic shocks on employment and real wages, social safety nets to shield vulnerable populations must be fortified. Policymakers should plan for expansion of unemployment benefits, implementation of wage subsidies, and provision of targeted assistance for industries severely affected by such crises to guarantee the well-being of citizens in tumultuous times.
(5) To bolster the economy’s resilience to future shocks, policymakers should promote economic robustness by fostering diversification across industries, investing in human capital development, and instituting stringent financial regulations to minimize the potential for economic fluctuations. These measures can aid in establishing a stable foundation for enduring growth.

The analysis presented in this study elucidates the complex interplay between shocks in energy price, energy efficiency, monetary policy, and the Korean economy during the turbulent era of the COVID-19 pandemic. However, this investigation is not without its limitations. In this study, we focus exclusively on the Korean economy. Therefore, the generalizability of our findings to other national contexts may be limited. The analysis is circumscribed by the availability and quality of the data, which may potentially have influenced the precision and validity of the results. We employ Bayesian estimation and impulse response functions, which, while robust, may not capture all the nuances of the complex relationships among the variables of interest. The investigation does not account for potential structural changes in the economy or the influence of other external factors that may have impacted relationships among economic indicators during the pandemic. The study’s timeframe is constrained to the COVID-19 pandemic period, thus precluding examination of the long-term effects of the identified shocks on the Korean economy.

We also propose five potential avenues for future research. The investigation could be expanded to encompass a comparative analysis of multiple economies and explore the generalizability of our findings across diverse contexts. Additional data sources and novel methodologies could be utilized to enhance the robustness and validity of the analysis. The potential moderating effects of other economic variables, such as fiscal policy, on the relationships between shocks in energy price, energy efficiency, monetary policy, and economic indicators, could be studied. Future researchers could investigate structural changes in the economy as well as the role of external factors in shaping the observed relationships during the pandemic. Finally, a longitudinal study could be conducted to assess the enduring effects of the identified shocks on the Korean economy and the persistence of these relationships beyond the COVID-19 pandemic period.
